# Childhood Trauma and Cortisol Reactivity: An Investigation of the Role of Task Appraisals

**DOI:** 10.3389/fpsyg.2022.803339

**Published:** 2022-04-11

**Authors:** Cory J. Counts, Annie T. Ginty, Jade M. Larsen, Taylor D. Kampf, Neha A. John-Henderson

**Affiliations:** ^1^Montana State University, Bozeman, MT, United States; ^2^Department of Psychology and Neuroscience, Baylor University, Waco, TX, United States

**Keywords:** childhood trauma and adversity, cortisol, stress, challenge and threat appraisals, blunted reactivity

## Abstract

**Background:**

Childhood adversity is linked to adverse health in adulthood. One posited mechanistic pathway is through physiological responses to acute stress. Childhood adversity has been previously related to both exaggerated and blunted physiological responses to acute stress, however, less is known about the psychological mechanisms which may contribute to patterns of physiological reactivity linked to childhood adversity.

**Objective:**

In the current work, we investigated the role of challenge and threat stress appraisals in explaining relationships between childhood adversity and cortisol reactivity in response to an acute stressor.

**Methods:**

Undergraduate students (*n* = 81; 61% female) completed an online survey that included general demographic information and the Risky Families Questionnaire 24 h before a scheduled lab visit. In the lab, a research assistant collected a baseline salivary cortisol sample. Following the baseline period, participants were read instructions for the Trier Social Stress Test (TSST), a validated psychological lab stressor. Next, they completed a challenge vs. threat task appraisal questionnaire and completed the speech and math portion of the TSST. Twenty minutes following the start of the TSST, a second salivary sample was collected to measure changes in salivary cortisol following the TSST.

**Results:**

Linear regression analyses adjusted for age, sex, childhood socioeconomic status (SES), and baseline cortisol levels, showed childhood adversity associated with changes in cortisol levels [*B* = –0.29 *t*(73) = –2.35, *p* = 0.02, *R*^2=0.07^]. Linear regression analyses controlling for age, sex, and childhood SES showed childhood adversity associated with both challenge [*B* = –0.52 *t*(74) = –5.04, *p* < *0.001, R*^2=0.24^] and threat [*B* = 0.55 *t*(74) = 5.40, *p* < 0.001, *R*^2=0.27^] appraisals. Significant indirect effects of childhood trauma on cortisol reactivity were observed through challenge appraisals [*B* = –0.01 (95% confidence interval = –0.02, –0.003)], and threat appraisals [*B* = –0.01 (95% confidence interval = –0.01, –0.003)].

**Conclusion:**

Childhood adversity may contribute to blunted cortisol reactivity, a pattern of response which is linked to obesity, addiction, and other behavior-related diseases. Our findings suggest that this relationship is in part a product of stress appraisals.

## Introduction

When individuals experience or perceive a stressor, the body produces a physiological response which is designed to aid in overcoming the stressor. This response involves activation of the hypothalamic-pituitary-adrenal (HPA) axis, releasing cortisol into the blood stream which promotes energy mobilization (Smith and Vale, 2006). The pattern and quantity of cortisol release in response to acute psychological stress varies across individuals, with some individuals displaying exaggerated or blunted responses. Exaggerated cortisol response is characterized by increases in cortisol levels that are greater than the average response which may include slower recovery to baseline. Previous research indicates that these exaggerated responses may leave an individual at higher risk for deleterious health effects such as chronic disease and early mortality through biological wear and tear on bodily systems (Lovallo, 2015). A separate body of research has shown that cortisol reactivity to stress may also be blunted or diminished, which is characterized as increases in cortisol which are below the average response. Blunted cortisol reactivity has previously associated with downstream health-relevant outcomes such as addiction, obesity, and depression (Lovallo, 2011; Phillips et al., 2013; al’Absi et al., 2021).

Differences in cortisol responses to stress have also been related to childhood adversity (Lovallo et al., 2012; Voellmin et al., 2015; Young et al., 2021). In prior work, childhood adversity was related to stress sensitization and vulnerability to future stressful events (Bunea et al., 2017) which may be hallmarked by dysregulation in HPA functioning characterized as increased sensitivity to stress and diminished physiological capacity to respond and combat stress (McEwen, 1998). Childhood adversity puts individuals at high risk for extreme and chronic levels of stress exposure during critical developmental stages (Ridout et al., 2018). Research suggests that cortisol responsivity may become diminished in response to chronic and elevated incidences of stress exposure (Fries et al., 2005) especially during early stages of brain development (VanTieghem and Tottenham, 2018). This hypothesis is highlighted in a body of research showing that individuals who report childhood adversity exhibit dysregulated stress reactivity characterized by diminished cortisol reactivity in response to a lab induced stressor (Carpenter et al., 2011; Voellmin et al., 2015; Bunea et al., 2017). In turn, these diminished cortisol responses to stress may explain the previously documented relationship between childhood adversity and chronic inflammation (for review; see Coelho et al., 2014).

The psychological mechanisms which may contribute to the documented relationship between childhood adversity and cortisol reactivity to stress are less understood. Prior research has shown that differences in the psychological appraisal of a stressor informs the pattern of physiological responses to stress. A large body of work on stress appraisals focuses on challenge (i.e., having adequate resources to meet the demand) compared to threat appraisals (i.e., not having adequate resources to meet the demand) (Uphill et al., 2019). Compared to challenge appraisals, threat appraisals have previously been related to increased reports of stress (Tomaka et al., 1993) and other poor cognitive, behavioral and affective responses to laboratory stressors (Drach-Zahavy and Erez, 2002; Jamieson et al., 2012). According to the biological embedding model, childhood is a period of heightened sensitivity and plasticity (Miller et al., 2011). The model posits that in addition to affecting the programming of biological systems, high levels of adversity in the childhood environment give rise to excessive vigilance for threat. As such, individuals who experience high levels of adversity during childhood may be more likely to appraise future events as threatening rather than challenging.

In past work, challenge and threat appraisals have also been found to have distinct profiles of cardiovascular activity in response to stress (Quigley et al., 2002). A threat profile, viewed as a less efficient cardiovascular response, is characterized by blunted cardiac output reactivity and increased vascular resistance compared to that which is observed in challenge states (Mendes et al., 2008). In previous work, threat appraisals were identified as a mediator in the relationship between depression and blunted systolic blood pressure (Brindle et al., 2013). Separately, previous literature indicates that cortisol and cardiovascular reactivity are linked to similar cortical and limbic regions of the brain (Gianaros and Wager, 2015; Gianaros et al., 2017; Ginty et al., 2017). Based on these findings, it is possible that as previously observed with cardiovascular reactivity (Brindle et al., 2013), threat appraisals of stressors may be linked to blunted cortisol reactivity.

It is currently unclear if differential perceptions of stress contribute to the association between childhood trauma and blunted cortisol reactivity. In the current work, we test our hypothesis that individuals who report higher levels of childhood adversity will exhibit blunted cortisol reactivity to stress, in part due to greater threat and lower challenge appraisals of an acute psychological stressor.

## Methods and Methods

### Procedure

Participants were college students enrolled in an introductory to psychology course at a 4-year state university. As part of the course requirements, students are asked to participate in research studies managed by the Psychology department. Participants were recruited through an online database in which they found and signed up for timeslots. All sessions were run during a 3-h time block (between 1 and 4 p.m) in order to control for time of day effects on cortisol levels. Twenty-four hours before their scheduled lab visit, participants who signed up were sent an online survey by email containing an informed consent form, general demographic information survey, and the Risky Families Questionnaire. Participants were required to complete the survey in order to participate in the lab portion of the study. Participants came to the lab during their scheduled lab visit and were seated in a chair. A research assistant asked them to limit their movement for a 10-min baseline period. During the last minute of the baseline period, participants provided a salivary sample for measurement of cortisol. A double tube salivary cortisol device (Salimetrics, CA, United States), was utilized to collect cortisol. The participant was instructed to remove a piece of cotton from the tube and chew on it for 60 s. The participant returned the piece of chewed cotton immediately to the collection tube upon completion.

Following the baseline period, all participants completed the Trier Social Stress Test (TSST), a validated social stressor, known to elicit changes in cortisol levels (Kirschbaum et al., 1993). Two research assistants were present in the room and evaluated participants for the duration of the TSST. One research assistant communicated all instructions for the task and answered any questions. The participant was seated for the duration of the TSST facing the two evaluators and a video camera that they believed was taping their performance. One research assistant explained the instructions for the Trier Social Stress Test (TSST) as follows, “You are being asked to complete a speech task detailing why you would be the ideal candidate for your dream job. The speech task will last for 5 min, and you will have the next 5 min to prepare for the speech. You will also complete a 5- min arithmetic task. Your performance will be videotaped and evaluated by a panel of judges trained in public speaking. Do you have any questions before we begin?” Once the research assistant answered any questions, the participant completed a brief stress appraisal measure and then began the 5-min speech task.

After the 5-min speech period, the research assistant read the instructions for a 5-min math task. The participant was given the following instructions: “You are now being asked to complete a math task. You will sequentially subtract the number 13 from 1,022. If you make a mistake, you will be asked to start over from 1,022. If you complete the sequence before time is up, please start over from 1,022”. The research assistant answered any questions before the participant began the task. At the conclusion of the 5-min period, a 10-min recovery period began during which the participant was asked to remain comfortably seated in their chair. At the end of the 10-min recovery period, the participant provided a second salivary cortisol sample using the method previously described. The research assistant concluded the study by explaining the true nature of the study and informed the participant that their performance was not actually being recorded.

### Measures

#### Childhood Adversity

We used the Risky Families Questionnaire (RFQ), a 13 item self-report measure to assess the degree of risk of physical, mental, and emotional adversity that an individual faced in their childhood and adolescent family environment (Taylor et al., 2004). Participants indicate how frequently certain events or situations occurred during the ages of 5–15 years using a 5-point likert scale (1 = not at all and 5 = very often). Example questions from this scale include, “How often did a parent or other adult in the household make you feel that you were loved, supported and cared for?” and “How often did a parent or other adult in the house push, grab, shove or slap you?” Items measuring the presence of positive qualities or experience were reversed scored and all 13 items were then summed to reflect overall risk in the family environment.

#### Challenge and Threat Appraisal

After research assistants described the upcoming TSST, all participants were provided with the following statement “A challenge state is experienced when an individual perceives they have sufficient, or nearly sufficient, resources to meet the demands of a task or situation, whereas a threat state is experienced when an individual perceives they have insufficient resources to meet the demands of a task or situation.” And then asked to indicate the degree to which they agreed with a series of 6 statements as a measure of their appraisal of the task as a challenge or as a threat (Williams et al., 2010; Trotman et al., 2018), adapted from McGregor and Elliot (2002). The statements which measured challenge appraisal were the following: “I view this task as a challenge,” “The task presents itself as a challenge to me,” and “I feel challenged by this task.” The statements used to measure task appraisals were identical apart from replacing the word “challenge” with “threat.” Participants rated the extent to which they agreed with each statement ranging from 1 (*not at all true*), to 7 (*very true*). The challenge and threat scales had adequate reliability, with Cronbach’s alpha being 0.81 and 0.83, respectively, which is in line with previous research using the scale (Williams et al., 2010; Trotman et al., 2018). Previous research has demonstrated that the challenge and threat subscales are independent of one another and not the same construct (Williams et al., 2010).

#### Cortisol

Upon collection, all salivary samples were immediately stored in a fridge and were transferred to a –80°C freezer located in the Stress, Adversity, Resilience and Health (SARAH) lab located on the Montana State University campus within 4 h of collection. Samples were thawed on the day of analyses and centrifuged at 1500 × *g* for 15 min. All samples were processed by trained SARAH lab research staff using a high sensitivity ELISA (Salimetrics, CA, United States). The inter-assay and intra-assay coefficient were below 8%. As we have done in previous research (John-Henderson et al., 2020), we calculated a cortisol difference score using the sample collected during baseline and the post-stressor sample (i.e., the sample collected 20 min after the start of the TSST).

#### Covariates

Based on previous research indicating relationships between biological sex, age, childhood socioeconomic status, oral contraceptive use, symptoms of depression and anxiety and cortisol reactivity to a social stressor (Kudielka et al., 2004; Liu et al., 2017; Lê-Scherban et al., 2018; Mazurka et al., 2018; Fiksdal et al., 2019; Gervasio et al., 2022), we measured these variables as covariates for our analyses. Participants self-reported their biological sex and age. As a measure of childhood socioeconomic status (SES), we used the MacArthur scale of subjective childhood SES, which is used to capture SES during childhood across objective SES indicators. Participants are presented with a 10-rung ladder and are asked to indicate where they feel their family stood during childhood relative to other families in the United States (Adler et al., 2000). Scores ranged from 1 (lowest SES) to 9 (highest SES). The question explains that the top of the ladder represents those families with more money, higher levels of education, and better jobs, while the bottom of the ladder represents those families with the lowest incomes, less education, and had low paying jobs or were unemployed. Female participants self-reported whether they were currently using oral contraceptives, and current symptoms of depression and anxiety were measured using the Hospital Anxiety and Depression scale (HADS; Zigmond and Snaith, 1983).

### Statistical Analyses

Analyses were conducted using SPSS (version 24; IBM, Armonk, NY, United States). Continuous covariates were centered before being used in analyses. A two-way repeated measures ANOVAs (baseline, 20 min after stress onset) using cortisol was conducted to determine if the stress task successfully perturbed the HPA axis. We tested our primary hypotheses using linear regression models. We examined relationships between childhood adversity and stress appraisals, stress appraisals and changes in levels of salivary cortisol, and the relationship between childhood adversity and changes in levels of salivary cortisol. Next, we explored potential indirect effects of childhood adversity on changes in levels of salivary cortisol. To test for indirect effects, following suggestions by Preacher and Hayes (2008), we used a bootstrapping approach in which a point estimate of the indirect effect was derived from the mean of 5000 estimates of the indirect pathways, and 95% confidence intervals were computed using the cutoffs for the 2.5% highest and lowest scores of the distribution. Indirect effects were considered statistically significant if the confidence interval did not include 0. For these analyses, demographics, oral contraceptive use, symptoms of depression and anxiety were used as covariates based on documented relationships between these measures and our outcome of interest as referenced earlier.

## Results

Descriptive statistics for the sample are listed in Table 1 and bivariate correlations between main variables of interest are listed in Table 2. A two-way repeated- measures ANOVA using cortisol activity during baseline (Mean = 0.36, Standard Deviation = 0.30) and 20 min after stress onset (Mean = 0.57, Standard Deviation = 0.37) demonstrated that the TSST significantly perturbed cortisol activity, *F*(1,80) = 62.02, *p* < 0.001, _*p*_eta^2^ = 0.437. Over 90% of participants showed an increase in cortisol activity from baseline to 20 min post stress onset. There were no statistically significant differences related to biological sex for reported levels of childhood adversity [*t*(79) = –0.62, *p* = *0.54*], reported challenge appraisals [*t*(79) = –0.74, *p* = *0.46*], or observed cortisol reactivity [*t*(79) = –1.05, *p* = *0.29*].

**TABLE 1:** Means and standard deviations.

Variable	*M*	*SD*	% Female
Gender			61.7%
Childhood socioeconomic status	3.12	1.23	
Age (years)	21.16	4.04	
Baseline cortisol (ug/dL)	0.36	0.30	
Post-task cortisol (ug/dL)	0.57	0.37	
Change in cortisol (ug/dL)	0.21	0.25	
Challenge appraisal average	3.30	1.54	
Threat appraisal average	2.50	1.58	
Risky family questionnaire	19.31	7.24	
Anxiety symptoms	20.53	3.35	
Depressive symptoms	10.03	3.96	

**TABLE 2:** Correlations.

Variable	1	2	3	4	5	6	7	8	9	10	11
1 Childhood socioeconomic status											
2 Age	–0.12										
3 Gender	0.16	−0.29**									
4 Baseline cortisol	0.09	0.04	0.02								
5 Post-task cortisol	0.02	0.12	0.10	0.75**							
6 Change cortisol	–0.07	0.13	0.12	–0.10	0.59**						
7 Challenge appraisal average	−0.24*	–0.10	0.08	–0.08	0.24*	0.46**					
8 Threat appraisal	0.27*	–0.11	0.15	0.14	–0.18	−0.43**	−0.60**				
9 Risky family questionnaire	0.18	–0.08	0.07	0.12	–0.09	−0.29**	−0.54**	0.57**			
10 Anxiety symptoms	–0.04	–0.07	0.05	0.13	0.16	08	0.24*	0.91	–0.22		
11 Depressive symptoms	–0.01	–0.10	0.08	–0.04	–0.08	–0.07	–0.08	0.85	–0.05	−0.40**	

** indicates correlation is significant at the 0.01 level (2-tailed), * indicates correlation is significant at the 0.05 level (2-tailed).

### Childhood Adversity and Stress Appraisals

We utilized a linear regression controlling for age, biological sex, childhood SES, and symptoms of depression and anxiety to examine relationships between childhood adversity (as measured by the Risky Family Questionnaire) with stress appraisals. We found that childhood adversity was positively related to threat appraisals [*B* = 0.55 *t*(74) = 5.40, *p* < 0.001, *R*^2=0.27^] and negatively related to challenge appraisals [*B* = –0.52 *t*(74) = –5.04, *p* < *0.001. R*^2=0.24^].

### Stress Appraisals and Changes in Salivary Cortisol

In a linear regression model controlling for age, biological sex, childhood SES, symptoms of depression and anxiety, oral contraceptive use and baseline levels of cortisol, challenge and threat appraisals were significant predictors of changes in levels of salivary cortisol [*B* = 0.48 *t*(73) = 4.61, *p* < 0.001, *R*^2=0.21^] and [*B* = –0.44 *t*(73) = –4.21, *p* < 0.001, *R*^2=0.18^], respectively.

### Childhood Adversity and Changes in Salivary Cortisol

Separately, in a linear regression model controlling for the same previously described covariates including baseline levels of cortisol, we examined the relationship between childhood adversity and changes in cortisol from baseline to after the TSST. Childhood adversity was negatively associated with changes in cortisol [*B* = –0.29 *t*(73) = –2.35 *p* = 0.02, *R*^2=0.07^], with greater reports of childhood adversity relating to smaller increases in levels of salivary cortisol.

Using the previously described method (Preacher and Hayes, 2008), with the same covariates described previously, and with the addition of each respective stress appraisal, we found a significant indirect effect of childhood adversity on changes in cortisol through challenge appraisals and threat appraisals. The bootstrapped unstandardized indirect effect of childhood adversity on cortisol reactivity through challenge appraisals was –0.01, and the 95% confidence interval ranged from –0.02 to –0.003. The bootstrapped unstandardized indirect effect of childhood adversity on cortisol reactivity through threat appraisals was –0.01, and the 95% confidence interval ranged from –0.01 to.003^1^. The direct effect of childhood adversity on cortisol reactivity when controlling for challenge appraisals was *B* = –0.001, *t*(77) = –0.26, *p* = 0.79, and the direct effect of childhood adversity on cortisol reactivity when controlling for threat appraisals was *B* = –0.002, *t*(77) = –0.45, *p* = 0.65.^2^

## Discussion

The findings reported here contribute to a significant body of work documenting relationships between childhood adversity and future patterns of cortisol responses to social stressors. In line with prior work (Carpenter et al., 2011; Lovallo et al., 2012; Voellmin et al., 2015; Bunea et al., 2017; al’Absi et al., 2021; Young et al., 2021; Brindle et al., 2022), our findings indicated a relationship between childhood adversity and cortisol reactivity such that individuals who reported more adversity during their childhood (age 5–15), had blunted cortisol reactivity compared to those individuals who reported less adversity.

Our findings extend previous work in this area by highlighting a potential psychological mechanism which may account for observed relationships between childhood adversity and cortisol responses to stress later in life. Specifically, our findings suggest that differences in challenge and threat stress appraisals linked to childhood adversity, may contribute to blunted cortisol responses. Specifically, individuals who reported more childhood trauma reported greater threat appraisals (i.e., not having adequate resources to meet demand) and lower challenge appraisals (i.e., having adequate resources to meet demand) compared to those who reported lower levels of childhood trauma. In previous research, threat appraisals have also been linked to poor behavioral, cognitive and affective responses to stress (Tomaka et al., 1993; Drach-Zahavy and Erez, 2002; Jamieson et al., 2012).

The present study is not without limitations. The second sample which was meant to reflect changes in salivary cortisol associated with completing the stressor, was collected at 20 min following the start of the task. While we observed a statistically significant increase in cortisol activity from baseline to this collection timepoint and over 90% of participants displayed an increase in cortisol from baseline to the collection timepoint, we may have missed the “peak” response time by collecting saliva too early. Methodological approaches to calculating stressor-evoked cortisol reactivity differ. Some work suggests calculating area under the curve (Pruessner et al., 2003), while other work suggests calculating a difference score between the maximum and minimum values (Miller et al., 2018). Nevertheless, in future work, multiple salivary samples should be collected over a longer time period in order to better understand how childhood trauma and related psychological appraisals of stressors map on to trajectories of salivary cortisol in response to a stressor. Additionally, the current sample was predominantly composed of non-Hispanic White college students. In future work, a more diverse sample would allow us to understand if the observed relationships are specific to this racial group or if they would differ in other racial and ethnic groups. In addition, simultaneous measurement of reactivity across multiple physiological systems would further develop our understanding of correspondence or divergence of responses across systems.

Prior work indicates that for females, the point in the menstrual cycle at the time of data collection could affect cortisol levels and cortisol reactivity. We did not collect this information in the current study and so were unable to account for the potential effect of this measure on our outcome. Finally, since we allowed for questions between the speech task and the math task of the TSST, there is some variability in the total time of the TSST across participants, however, this variability should be very limited (i.e., under 1 min).

Overall, the findings presented here raise the possibility that stress appraisals may contribute to the link between childhood trauma and cortisol responses to stress. As noted previously, the blunted pattern of cortisol response, which was linked to low challenge appraisals, high threat appraisals, and high reports of childhood trauma, is related to a host of behavioral outcomes which in turn relate to poor mental and physical health outcomes.

Previous work demonstrates the feasibility of manipulating or directing stress appraisals toward either challenge or threat, and provides evidence that these distinct appraisals correspond with unique physiological responses to the stressor (John-Henderson et al., 2015; Williams et al., 2017). As such, it is possible that interventions which aim to reshape stress appraisals (i.e., toward challenge) for individuals who report high levels of childhood trauma may reduce risk for chronic health conditions and behavioral outcomes which are linked to blunted cortisol reactivity. Finally, with regards to implications of our findings, as discussed previously, blunted cortisol reactivity is related to obesity, addiction, and depression (Phillips, 2011; Phillips et al., 2013; al’Absi et al., 2021). It is possible that psychological interventions which work to reshape stress appraisals (i.e., toward challenge rather than threat) may increase cortisol reactivity to social stressors in individuals who experienced childhood adversity, and in doing so could reduce the high incidence of obesity, addiction, and depression observed in this population.

## Data Availability Statement

The raw data supporting the conclusions of this article will be made available by the authors, without undue reservation.

## Ethics Statement

The studies involving human participants were reviewed and approved by Montana State University Institutional Review Board. The patients/participants provided their written informed consent to participate in this study.

## Author Contributions

CC designed the study, collected the data, analyzed the data, wrote the manuscript, and helped with revisions. AG helped with the conceptual framing of the manuscript, data analyses and interpretation, and manuscript revisions. JL and TK helped with the literature review, table creation, and manuscript revisions. NJ-H helped design the study and helped with data analyses and with writing and revision of the manuscript.

## Conflict of Interest

The authors declare that the research was conducted in the absence of any commercial or financial relationships that could be construed as a potential conflict of interest.

## Publisher’s Note

All claims expressed in this article are solely those of the authors and do not necessarily represent those of their affiliated organizations, or those of the publisher, the editors and the reviewers. Any product that may be evaluated in this article, or claim that may be made by its manufacturer, is not guaranteed or endorsed by the publisher.

## Footnotes

1 Based on previously documented relationships between biological sex and cortisol reactivity patterns, we tested whether biological sex moderated the statistically significant mediation we reported here. Biological sex was not a statistically significant moderator of this mediation model.

2 We conducted sensitivity analyses for which we excluded two outliers who were 3 standard deviations above the average age of our sample. The pattern of our findings did not change after excluding these outliers.
